# Corrigendum to: Current state of scientific evidence on Internet-based interventions for the treatment of depression, anxiety, eating disorders and substance abuse: an overview of systematic reviews and meta-analyses

**DOI:** 10.1093/eurpub/ckab104

**Published:** 2021-07-07

**Authors:** C Barr Taylor, Andrea K Graham, Rachael E Flatt, Karin Waldherr, Ellen E Fitzsimmons-Craft

**Affiliations:** 1 Center for m^2^Health, Palo Alto University, Palo Alto, CA, USA; 2 Department of Psychiatry & Behavioral Sciences, Stanford University, Palo Alto, CA, USA; 3 Department of Medical Social Sciences, Northwestern University, Chicago, IL, USA; 4 FernFH Distance Learning University of Applied Sciences, Wiener Neustadt, Austria; 5 Department of Psychiatry, Washington University School of Medicine, St. Louis, MO, USA

*European Journal of Public Health*, 2019; doi:10.1093/eurpub/ckz208

In the originally published version of this article, there was an error in the corresponding author’s name. This should read: “C Barr Taylor” instead of “C Taylor”. The error has now been corrected.

