# Supplementary material for: Current state of scientific evidence on Internet-based interventions for the treatment of depression, anxiety, eating disorders and substance abuse: an overview of systematic reviews and meta-analyses
Source: Eur J Public Health. 2020 Sep 11;31(Suppl 1):i3–i10. doi: 10.1093/eurpub/ckz208 (PMC8495688; doi:10.1093/eurpub/ckz208)
Supplement: ckz208_Supplementary_Data [file ckz208_supplementary_data.zip › ckz208-suppl_data/Current State of InternetFINAL10.2.19.docx]

**Current state of scientific evidence on Internet-based interventions for the treatment of depression, anxiety, eating disorders, and substance abuse: An overview of systematic reviews and meta-analyses**

C. Barr Taylor^1,2^, Andrea K. Graham^3^, Rachael E. Flatt^1,2^, Karin Waldherr^4^, & Ellen E. Fitzsimmons-Craft^5^

^1^Center for m^2^Health, Palo Alto University, Palo Alto, CA, USA

^2^Department of Psychiatry & Behavioral Sciences, Stanford University, Palo Alto, CA, USA

^3^Department of Medical Social Sciences, Northwestern University, IL, USA

^4^FernFH Distance Learning University of Applied Sciences, Wiener Neustadt, Austria

^5^Department of Psychiatry, Washington University School of Medicine, St. Louis, MO, USA

Correspondence: C. Barr Taylor, Palo Alto University, 1791 Arasstradero Rd, PaloAlto , Ca, USA, 94304. Email: btaylor@stanford.edu

**Abstract**

**Background**

ICare represents a consortium of European Investigators examining the effects of online mental health care for a variety of common mental health disorders provided in a variety of settings. This paper provides an overview of the evidence of effectiveness for Internet-based treatment for four common mental health disorders that are the focus of much of this work: depression, anxiety, substance abuse, and eating disorders.

**Methods**

The overview focused primarily on systematic reviews and meta-analyses identified through PubMed (Ovid) and other databases and published in English. Given the large number of reviews specific to depression, anxiety, substance abuse, and/or eating disorders, we did not focus on reviews that examined the effects of Internet-based interventions on mental health disorders in general. Each article was reviewed and summarised by one of the senior authors, and this review was then reviewed by the other senior authors. We did not address issues of prevention, cost-effectiveness, implementation, or dissemination, as these are addressed in other reviews in this supplement.

**Results**

Across Internet-based intervention studies addressing depression, anxiety, substance abuse, and eating disorders primarily among adults, almost all reviews and meta-analyses found that these interventions successfully reduce symptoms and are efficacious treatments. Generally, effect sizes for Internet-based interventions treating eating disorders and substance abuse are lower compared to interventions for depression and anxiety.

**Conclusion**

Given the effectiveness of Internet-based interventions to reduce symptoms of these common mental health disorders, efforts are needed to examine issues of how they can be best disseminated and implemented in a variety of health care and other settings.

**Keywords:** Internet-based interventions, depression, anxiety, substance abuse, eating disorders

**Introduction**

The psychological treatment of mental health problems is undergoing a radical transformation, driven by the near universal availability of digital technology and daily use of digital technology by most people for a variety of functions and activities^1^. By digital technology we refer to the use of computers, the Internet, mobile devices, apps, and similar digital devices often used in combination. These technologies are provided as stand-alone self-help programs, guided/moderated programs, or blended programs with some face-to-face encounters, and/or in various combinations^2^. Digital interventions are seen as advantageous to other psychological modes of delivery because they are easily accessible, private (thus avoiding some issues of stigma), potentially more affordable, and can be readily personalised and tailored to individual needs and interests^3^.

Given the importance of digital technology to transform mental health services, the aim of the EU-funded consortium ICare (Integrating Technology into Mental Health Care Delivery in Europe) wass to establish an online platform that provides evidence-based interventions for the prevention and treatment of the most prevalent mental health problems and disorders, to implement the interventions into real-world contexts in Europe, to evaluate their effectiveness and cost-effectiveness, and to study moderators and mediators of intervention outcomes (www.icare-online.eu). As an introductory article in this supplement, we summarise the current state of knowledge in relation to the clinical trials on Internet-based treatments for four of the common mental health disorders included in ICare: depression, anxiety, substance abuse, and eating disorders. More specifically, the aim is to present a brief overview of recent systematic reviews and meta-analyses that have evaluated the effectiveness of Internet-based treatments for these disorders primarily among adults. Based on these studies, we also identify important issues and emerging directions for future research. Consistent with the goals of ICare, we anticipated that findings from the meta-analyses and systematic reviews would support research that continues to examine issues related to the effectiveness of Internet-based interventions as provided to large populations in a variety of settings.

**Methods**

We began by conducting a search of PubMed (Ovid), PsychInfo, and Embase. The searches were limited to English language articles published in the last six years. Our search focused on Internet-based interventions that were tested primarily among adults. The search terms included: systematic reviews and meta-analyses of Internet, mobile/smartphone and/or web-based treatment of depression, anxiety, substance abuse, and eating disorders. We also conducted searches focusing on specific anxiety disorders: post-traumatic stress disorder (PTSD), social phobia, generalised anxiety disorder (GAD) and obsessive-compulsive disorder (OCD). We did not include articles on tobacco use as this is not a focus of ICare. Articles identified were reviewed from most recent backwards, with a forward search also conducted for the most recent articles. Articles were accepted if they (a) included information relevant to the topics of the current overview (Internet-based intervention for depression, anxiety, substance abuse and/or eating disorders), (b) were a published meta-analysis, systematic review, or rapid review, (c) focused mostly on randomised trials, (d) included RCTs where participants were primarily adults, and (e) were in English. Specifics regarding the different types of comparator interventions are included in the individual sections. Given the large number of reviews specific to depression, anxiety, substance abuse, and/or eating disorders, we did not focus on reviews that examined the effects of Internet-based interventions on mental health disorders in general. Each article was reviewed and summarised by one of the senior authors (CBT, AKG, or EFC), and this review was then reviewed by the other senior authors. ICare also includes interventions for adjustment disorders, but we found no meta-analyses or systematic reviews that met our criteria, so this disorder was not included below.

One of the problems of this approach is that many of the reviews and meta-analyses report on the same articles. For this reason, as mentioned above, we focused on the most recent articles. We did not address issues of prevention, cost-effectiveness, implementation, or dissemination, as these are addressed in other reviews in this special issue.

**Results**

*Depression*

We initially identified 339 different articles (not including abstracts), of which 14 met our search criteria. Of the 14, one study was omitted because it did not include detailed statistical data, another because the review included a number of non-Internet-based interventions, and a third because most of the interventions were quasi-experimental or included several disorders or primarily adolescents, leaving 11 trials.

The most recent review for treating clinical depression included in our search was an update from a previous meta-analysis conducted in 2010^4^. The review followed PRISMA guidelines. In all, 4,423 abstracts were examined from a variety of searches, eventually leading to 53 studies in the meta-analysis. Only randomised controlled trials of Internet-based interventions versus either waitlist control, information control, care as usual, or placebo were included (see Supplementary Table 1 for more details on comparators). According to the authors, the control group was usually a delayed treatment group in which there was no expectation that the delay before treatment would be beneficial. Hedges’ effect sizes were calculated, and the number needed to treat (NNT) was also determined. Adherence, patient satisfaction, and bias risk were also assessed. In total, there were 32 depression studies involving 5,642 subjects. The overall g-value at follow-up was 0.67 (95% CI = [0.51, 0.81]; P = 0.00), almost all using Internet-based cognitive-behavioral therapy (iCBT); NNT was 2.78. When compared with usual care, effect sizes were smaller but still significant. Adherence ranged from 16% to 100%, with an overall adherence (including anxiety studies) of 75% and an overall satisfaction of 85%. Follow-up effect sizes compared to immediately after trial completion, again combined for both depression and anxiety, were g = 0.15 (95% CI = [0.06, 0.23]; P = 0.05) at three to six months post treatment and g = 0.22 (95% CI = [0.01, 0.43]; P = 0.00) at nine to 18 months post treatment. Overall, when compared to face-to-face treatment, the effect size was g = 0.14 (95% CI = [-0.04, 0.32]), a non-significant difference. The authors concluded that iCBT was effective.

Wright et al.^5^ identified 40 randomised iCBT depression trials that met their inclusion criteria. The control groups were waitlist, attention control, or treatment as usual. The study included adolescent participants, if they were 16 years or older, but most included participants who were adults. The random effects weighted mean effect size for iCBT versus controls at post-treatment was g= 0.50 (95% CI = [0.39, 0.61]; P < 0.001). The random-effects weighted mean effect size for studies using the support of a coach was g = 0.67 (95% CI = [0.55, 0.80]; P < 0.001). By contrast, iCBT studies that did not include support had a random-effects weighted mean effect size at post-treatment of g = 0.24 (95% CI = [0.12, 0.36]; P < 0.001). Regarding effect sizes depending on the level of support provided, the random-effects weighted mean effect size at post-treatment was lowest for e-mail (n = 9; g = 0.56; 95% CI = [0.35, 0.94]; P < 0.001), intermediate for telephone (n = 9; g = 0.78; 95% CI = [0.56, 1.01]; P< 0.001), and largest when face-to-face person support was provided (n = 3; g = 0.83; 95% CI = [0.43, 1.24]; P < 0.001). The authors concluded that Internet-based interventions for depression are effective, but that self-guided interventions are considerably less effective.

Josephine et al.^6^ identified 19 controlled studies that reported on 29 different Internet- and mobile-based interventions for depression. The intervention had to be compared to another intervention or to one of the following controls: 1) no psychological treatment, 2) attention or psychological placebo, 3) waitlist control, or 4) active, non-Internet-based treatment. At the end of treatment, Internet-based interventions resulted in improved depression severity compared to waitlist conditions (g = -0.90 (95% CI = [-1.07, -0.73]. (If p values are not included in this overview, they were not reported by the authors.) Comparisons between different interventions did not demonstrate any superiority or inferiority, and heterogeneity among the studies was high. Depression symptoms were reduced across all interventions from pre- to post-treatment (within group g range = [-2.24, -0.64]) and from pre-treatment to follow-up (g range = [-3.07, -0.93]).

Karyotaki et al.^7^ examined the effects of self-guided iCBT for treatment of depressive symptoms using data from 3,876 participants from 13 of 16 studies they identified. Control conditions were attention placebo, no treatment, treatment as usual, or waiting list. Self-guided iCBT was significantly more effective than controls for reducing depressive symptoms severity (β = -0.21; Hedges g  = 0.27; 95% CI = [0.17, 0.37]; P < 0.001). In a subsequent study, Karyotaki et al.^8^ examined the efficacy of guided iCBT for depression compared to control groups. They identified 27 relevant randomised controlled trials (RCTs) and were able to obtain the data from 24, which then comprised of 4,889 participants. Treatment outcomes on response and remission were examined with mixed-effects models with participants nested within studies. The Reliable Change Index was used to calculate response and remission. The intervention group obtained significantly higher response rates (Odds Ratio (OR) = 2.49; 95% CI = [2.17, 2.85]; P < 0.001), and remission rates compared to controls (OR = 2.41; 95% CI = [2.07, 2.79]; P < 0.001). Adults with more severe depressive symptoms at baseline were more likely to remit after receiving treatment (OR = 1.19: 95% CI [1.01, 1.39]; P = 0.04). The results suggest that guided Internet-based interventions lead to substantial positive effects on treatment response and remission at post-treatment. The authors argue that an important finding was that guided self-help is useful for patients with more severe depression. They also note that, depending on the criteria, between 44% and 61% of the participants did not show response, and between 58% and 62% did not achieve remission.

Wahle et al.^9^ reviewed 45 studies, selected from 6,387 initial records that used various types of technology-based interventions to reduce depressive symptoms. Approximately 80% of the studies were based on CBT. The interventions showed a trend toward reduced depressive symptoms (SMD = –0.58; 95% CI = [–0.71, –0.45]; P < 0.001), but the findings were limited by the significant heterogeneity among trials. The authors also provided descriptive data on 15 components that were part of the trials. About 43% used homework, and about 25% used email reminders.

Twomey et al.^10^ undertook a meta-analysis of one depression program (Deprexis) that had been studied in eight trials with a combined sample of 2,402 participants. The authors selected only randomised controlled trials and “waiting list (or delayed treatment)” were used in all studies. Deprexis includes ten sessions, and the user can select which modules to use. The program demonstrated a medium effect size (g = 0.54; 95% CI = [0.39, 0.69]. The authors describe the program as “broadly based on CBT” but also describe it as including acceptance, mindfulness, attachment, interpersonal process, positive psychology, dream-work, nutrition, and physical exercise. Because users could select the order of components, and the effects of the components were not examined, it is not clear how much evidence this provides for the effects of iCBT per se.

As part of a broad review of Internet-delivered psychological treatments for mood and anxiety disorders, Arnberg et al.^11^ summarised the results of five iCBT randomised trials that included a waitlist control group that were identified as part of their search. The test for overall effect was Z = 6.75; SMD = 0.83; 95% CI = [0.59, 1.07], P < 0.001). They also noted that many of these studies were conducted with well-educated and employed populations, which raises concerns about their effects for less resourced populations, the lack of adequately powered noninferiority trials, the lack of information on implementation, and the paucity of information on long-term effects and information on children. Several articles that cite this publication have addressed some of these issues: for example, Thase et al.^12^ found that computer-assisted CBT that blends Internet-delivered skill-building modules with about five hours of therapeutic contact was noninferior to a standard course of CBT (up to 20 face-to-face sessions of 50 minutes each) that provided over eight additional hours of therapist contact. Klein et al.^13^ did not find differences in depression outcome measures.

Păsărelu et al.^14^ conducted a meta-analysis examining only transdiagnostic/tailored iCBT for adult anxiety and/or depression with control groups (i.e., waitlist comparison groups or active waitlists, such as attention modification or online discussion groups). The study included 19 randomised trials, and 2,952 participants that met inclusion criteria were included in the analyses. For uncontrolled effects (Hedges' g), transdiagnostic/tailored iCBT had large and medium effects for anxiety and depression outcomes and for quality of life, respectively. For controlled effects, iCBT had medium to large effect sizes for anxiety and depression outcomes (anxiety: g = 0.82; 95% CI = [0.58, 1.05], P = 0.00; depression: g = 0.79; 95% CI = [0.59, 1.00], P = 0.00) and medium effect sizes for quality of life (g = 0.56; 95% CI = [0.37, 0.73], P = 0.00). Of note, only two studies appeared to include participants with major depressive disorder. The authors concluded that transdiagnostic and tailored iCBT are effective for addressing anxiety and depression. (We did not include in the supplementary table because of the heterogeneity of the subject selection characteristics).

Richards & Richardson’s^15^ review included 40 studies and 19 RCTs; these studies were drawn from 45 and 23 published papers, respectively. The nature of the control groups was not explicitly specified, but based on tables in the paper, appeared to include waitlist, treatment as usual, and active treatment control groups. Across the 19 randomised studies, a moderate post-treatment pooled effect size was found (d = 0.56; 95% CI = [−0.71, −0.41]; Z = 7.48; P *<* 0.001). Therapist supported studies had a mean post-treatment effect size of d = 1.35 and d = 1.29 at follow-up. For coached or guided studies, the mean post-treatment effect size was d = 0.95 and at follow-up was d = 1.20 (P values were not reported for any of these effect sizes). Among studies in which no support was provided, the mean post-treatment effect was d = 0.78 and at follow-up was d = 1.13. Across 40 studies, an overall rate of participant dropout was 57%. By intervention type, dropout rates were 74% for no support (self-help), 38% for guided/coached, and 28% for direct therapy support. Thus, the authors concluded that supported interventions yielded better outcomes and had greater retention.

Hedman et al.^16^ evaluated 20 RCTs, which included over 4,776 patients with depression. Control groups were not specified but based on the table, included such conditions as online information about depression, treatment from a general practitioner, and face-to-face CBT. They found large treatment effects for iCBT for depression or depressive symptoms (d = 0.94; 95% CI = [0.77, 1.11]). They also found that in comparing iCBT to conventional CBT across a number of problems, treatment effects were equivalent for iCBT (d = 1.04; 95% CI = [0.73, 1.35]) and conventional CBT (d = 1.14; 95% CI = [0.72, 1.56]) in 12 RCTs.

There were additional articles of relevance to this review that did not meet our formal criteria, but we felt the results of two were worth including. In an effort to compare face-to-face CBT versus iCBT for “psychiatric and somatic conditions”, Carlbring et al.^17^ identified 20 studies that included 1,418 participants comparing face-to-face CBT with iCBT within the same trial. The pooled effect size at post-treatment was Hedges’ g = 0.05 (95% CI = [-0.09, 0.20]), indicating that the overall effects of iCBT and face-to-face treatment were equivalent. Karyotaki et al.^18^ included participant data obtained from 13 RCTs in a trials meta-analysis that examined potential “harmful” effects of iCBT. Control groups included waiting list, treatment as usual, attention placebo, or other non-active controls. There was a clinically significant deteriorate of 5.8% in the treatment groups compared to 9.1% in the control groups, a significant difference (OR = 0.62; 95% CI = [0.46, 0.83]; P < 0.001) compared with control conditions.

Innovations & Emerging Directions for Depression Interventions

In reviewing the literature, it became apparent that a number of Internet-based approaches and strategies to improve delivery and/or engagement are being developed and that some might improve outcomes of iCBT-based programs. Papers describe the use of games^19,20^, text messaging^21,22^, ecological momentary assessment^23^, and automated systems, including conversational agents^24^. Of these, text messaging and ecological momentary assessment systems have been widely used. We could not find reviews or meta-analyses that related these approaches for treating depression specifically. A noticeable gap in the literature is papers that examine the use of social media, peer support groups, and other interventions that might enhance outcomes.

Summary

Systematic reviews and meta-analyses support the efficacy of Internet-based interventions for treating depression and depressive symptoms, at least in adults, and in populations selected from higher socioeconomic demographics. Effect sizes are often in the moderate to strong range and are clinically important. Notably, the effectiveness of Internet-based interventions for depression has been demonstrated in a number of countries, with somewhat less evidence from studies in the U.S. High dropout rates and poor adherence are common. Few studies examine long-term effects, but short-term effects appear more substantial than longer ones. Online interventions are heavily dominated by iCBT-derived approaches. With some exceptions, therapist-supported or guided self-help intervention seem more effective than self-help. A few studies have found iCBT to be as effective as face-to-face treatment. More information is needed on the benefits of tailoring and providing single focused interventions^22^, component analysis, systematic integrated approaches to defined populations^23^ , and the effects on subtypes of depression and suicidality, to name a few topics. Little is known about the use of these techniques in low- and middle-income countries. Finally, more information is needed on the benefits of including social media, texting, and other synchronous and asynchronous activities.

**Anxiety**

We initially identified 339 different articles (not including abstracts). Of these, eight met our search criteria and are included in this review. Anxiety disorders encompass a number of different disorders, yet most of the meta-analyses combined intervention effects across a number of different interventions. We also conducted searches focusing on specific anxiety disorders: PTSD, social phobia, GAD, OCD and simple phobias. (We include studies in the supplementary table that present data on studies that focus on symptoms and overall effect on disorders, and studies specific to panic disorder/agoraphobia, social phobia, generalised anxiety disorder, and PTSD).

Andrews et al.^4^ identified 12 studies addressing panic disorder and agoraphobia, 11 studies addressing social anxiety disorder, and 9 studies addressing generalised anxiety disorder. All studies compared the Internet-based intervention to a control group including care as usual, waitlist, information control, psychological placebo, or pill placebo, among others. In terms of between-group effect sizes, for panic disorder, Hedges’ g was 1.31 (95% CI = [0.85, 1.76]; P < 0.001). For social phobia, Hedges’ g was 0.92 (95% CI = [0.76, 1.08]; P = 0.05). For generalised anxiety disorder, Hedges’ g was 0.70 (95% CI = [0.39, 1.01]; P < 0.001). The combined results for depression and anxiety are reported above.

Păsărelu et al.’s^14^ meta-analysis, which combined interventions for anxiety and depression, also reviewed above, found medium to large controlled effect sizes for anxiety (g = 0.82; 95% CI = [0.58, 1.05], P = 0.00).

Olthuis et al.^27^ identified 38 RCTs including 3,214 participants of therapist-supported iCBT compared to a waiting list, attention control, information, or online discussion group; unguided CBT (self-help); or face-to-face CBT. The studies examined social phobia (11 trials), panic disorder with or without agoraphobia (eight trials), generalised anxiety disorder (five trials), post-traumatic stress disorder (two trials), obsessive-compulsive disorder (two trials), and specific phobia (two trials). Eight remaining studies included a range of anxiety disorder diagnoses. The investigators made three primary comparisons: experimental versus waiting list control, therapist supported iCBT versus unguided iCBT, and experimental versus face-to-face CBT. Evidence from nine studies (644 participants) contributed to a pooled risk ratio (RR) of 4.18 (95% CI = [2.42, 7.22]) for clinically important improvement in anxiety at post-treatment, favoring therapist-supported iCBT over a waiting list, attention, information, or online discussion group only. Similarly, the SMD for disorder-specific symptoms at post-treatment evidence from 22 studies (n = 1573; SMD = -1.12; 95% CI = [-1.39, -0.85]) and general anxiety symptoms at post-treatment (14 studies; n = 1004; SMD = -0.79; 95% CI = [-1.10, -0.48]) favored therapist-supported iCBT. At post-treatment there were no clear differences between unguided CBT and therapist-supported iCBT for disorder-specific anxiety symptoms (four studies; n = 253; SMD = -0.24; 95% CI = [-0.69, 0.21]) or general anxiety symptoms (two studies; n = 138; SMD = 0.28; 95% CI = [-2.21, 2.78]). Compared to face-to-face CBT, therapist-supported iCBT showed no significant differences in clinically important improvement in anxiety at post-treatment (four studies; n = 365; RR = 1.09; 95% CI = [0.89, 1.34]). There were also no clear differences between face-to-face and therapist supported iCBT for disorder-specific anxiety symptoms at post-treatment (six studies; 424 participants; SMD = 0.09, 95% CI = [-0.26, 0.43]; low quality evidence) or general anxiety symptoms at post-treatment (five studies; 317 participants; SMD = 0.17; 95% CI = [-0.35, 0.69]). They concluded that therapist-supported iCBT appears to be an efficacious treatment for anxiety in adults and that therapist-supported iCBT is more efficacious than waiting list, information only, or online discussion groups. They also suggested that therapist-supported iCBT may not be inferior to face-to-face CBT in reducing anxiety.

Arnberg et al.^11^ identified nine trials that examined effects of iCBT on anxiety disorders. Eight trials evaluated the effect of therapist-guided iCBT compared to a waitlist control on social phobia (of note, five of the trials were interventions conducted by two research groups), with the overall effect being d = 0.85 (95% CI = [0.66, 1.05]; P < 0.001). They identified four trials for generalised anxiety disorder, with the overall effect being d = 0.84 (95% CI = [0.45, 1.23]; P < 0.001). Because of study limitations, small samples, and lack of controls, the authors were unable to draw conclusions about online interventions for panic disorder, specific phobias, posttraumatic stress disorder, obsessive-compulsive disorder or transdiagnostic approaches.

Hedman et al.^16^, also reviewed above in the depression section, looked at 103 RCTs, with over 12,374 participants with anxiety and depression. Large treatment effects were reported for iCBT for panic disorder (n = 407; d = 1.42; 95% CI = [0.86, 1.99]; nine RCTs), social phobia (n = 1448; d = 1.13; 95% CI = [0.99, 1.28]; 16 RCTs), post-traumatic stress disorder (n = 148; d = 1.23; 95% CI = [0.83, 1.63]; six RCTs), generalised anxiety disorder (n = 145; d = 1.12; 95% CI = [0.61, 1.62]; two RCTs), and transdiagnostic treatments for anxiety disorders (d = 1.07; 95% CI = [0.75, 1.39]; six RCTs). Individual studies on obsessive-compulsive disorder, severe health anxiety, and spider phobia also reported large treatment effects.

A meta-review of 11 systematic reviews and meta-analyses about the efficacy of Internet-based psychological treatments for anxiety disorders noted that there is general agreement on the efficacy of Internet-based psychological treatment as compared with non-treatment groups (with large effect sizes), finding similar efficacy compared with face-to-face therapies and improved when combined with some therapist contact^28^ .

Specific Anxiety Disorders

A few meta-analyses focused on specific anxiety disorders. Kampmann et al.^29^ conducted a meta-analysis to determine the efficacy of technology-assisted interventions for individuals with social anxiety disorder. They found 37 RCTs, enrolling 2,991 participants that were enrolled into iCBT trials (21 studies); the other studies used virtual reality exposure therapy (VRET). Patients undergoing iCBT showed significantly less social anxiety disorder symptoms at post-assessment than passive control conditions (g = 0.84; 95% CI = [0.72, 0.97]; P < 0.001). iCBT had a small advantage compared to active control conditions at post-assessment (g = 0.38; 95% CI = [0.13, 0.62]; P < 0.01). Sijbrandij et al.^30^ conducted a meta-analysis of 12 RCTs focused on PTSD. The pooled effect size of the 11 comparisons (10 studies, 1,139 participants) that compared iCBT to waitlist support or treatment as usual was moderate (g = 0.71; 95% CI = [0.49, 0.93]; P < 0.001). Only three studies compared iCBT to other interventions, and the effect size was small (g = 0.28; 95% CI = [0.00, 0.56]; P = 0.05).

Summary

iCBT for generalised anxiety disorder, social phobia, and PTSD is effective with at least moderate effect sizes. iCBT also seems effective for panic disorder. The results are less clear for obsessive-compulsive disorder.

**Substance Use Disorders**

Our search terms for this section included addiction, alcohol or drug abuse or dependence, Internet, systematic reviews, meta-analyses. We did not include interventions for tobacco use cessation, as this is not a primary focus of ICare. We identified 370 articles of potential interest and included four that met our criteria related to alcohol and two on other substances.

Alcohol

Kaner et al.^31^ examined personalised digital interventions for reducing hazardous and harmful alcohol consumption in community-dwelling populations. The authors identified 57 randomised studies that included a total of 34,390 participants. Overall, they found that participants enrolled in a digital intervention drank significantly less alcohol (22.8 g/week) (95% CI = [15.4, 30.3]) than participants who received no or minimal interventions at end of follow-up. When compared to controls, they found a mean reduction of up to three drinks per week. Of the five studies included in the review that compared digital interventions to in-person interventions, all found that both digital and in-person interventions produced no significant difference post-treatment. The authors concluded that there is “moderate” quality evidence that digital interventions may lower alcohol consumption.

Prosser et al.^32^ found 23 randomised controlled studies (n = 7,614) that examined the effects of digital interventions in reducing drinking in college/university students located in North America (Canada and U.S.) and Europe (the Netherlands, U.K., and Sweden). They found a small but significant effect in reduction of drinking (Z = 4.80; SMD = -0.15; 95% CI = [-0.21, -0.09]; P < 0.001). Interventions that included a personalised feedback component (such that participants were provided tailored feedback based on their level of consumption during the week) were significantly more efficacious than those interventions that did not.

Black et al.^33^ examined 93 randomised controlled trials examining the effectiveness of self-directed digital interventions in terms of reducing alcohol consumption against assessment-only control groups. The primary purpose of the study was to examine behaviour change techniques, but they did note that a meta-analysis of the studies found small, but significant effects on alcohol consumption, averaging across time points. The smallest pooled effect size was on heavy episodic drinking frequency (d = 0.07; 95% CI = [0.04, 0.10]) and the largest pooled effect size was on total consumption (d = 0.15; 95% CI = [0.11, 0.18]) (P values were not reported). The authors also found that smaller effects were associated with interventions that provided participants with information on consequences of alcohol consumption.

Finally, Riper et al.^34^ conducted a meta-analysis on 16 randomised controlled trials (with 23 comparisons and 5,612 participants) of both guided and unguided interventions for alcohol abuse. Control conditions included assessment-only, waitlist, or a psychoeducational brochure. Internet-based interventions were associated with a small but significant overall effect size on reducing alcohol consumption (g = 0.20; 95% CI = [0.13, 0.27]; P < 0.001), with women demonstrating the greatest reductions. While the effect size for guided interventions (g = 0.23) was slightly larger than for unguided interventions (g = 0.20), the authors found no significant difference between the two, possibly as a result of being underpowered and/or participant characteristics.

Other substance abuse

Boumparis et al.^35^ conducted a systematic literature search to identify RCTs comparing Internet interventions to control conditions in terms of their effectiveness in reducing the use of opioids, cocaine, and amphetamines. They identified 17 studies with 2,836 participants. Of these, four studies found that Internet interventions significantly decreased opioid use at post-treatment (n = 606; g = 0.36; 95% CI = [0.20, 0.53]; P < 0.001), and nine studies found reductions in illicit substance use at post-treatment (g = 0.35; 95% CI = [0.24, 0.45]; P < 0.001). Four studies found no significant effects of Internet interventions for stimulant users. Meanwhile, Tait et al.^36^ identified ten studies involving 4,125 participants that examined the effects of Internet or computer interventions on reducing the frequency of cannabis use compared to primarily assessment-only or informational control groups. They found a small, but significant overall effect size of g = 0.16 (95% CI = [0.09, 0.22]; P < 0.001) at post-treatment. They did not find significant differences between guided and unguided interventions, and there was no effect of number of sessions of the intervention on mean effect size.

Summary

Several meta-analyses and systematic reviews suggest that Internet-based interventions can have a small effect size on reducing alcohol consumption, at least in the short term. We could not find evidence that such interventions promote alcohol abstinence, and the data is strongest for college and university populations compared to other populations. Fewer studies have addressed the effects of Internet interventions on other types of substance abuse. There is also encouraging data suggesting that Internet interventions may have a small but significant effect on cannabis use.

**Eating Disorders**

Finally, our search for articles on eating disorders returned 240 papers, and seven met our search criteria and are included in this review.

A recent systematic review was conducted on mobile interventions for eating disorders^37^, and 15 studies of any design (e.g., experimental, observational) were reviewed. The interventions were evaluated as the sole means of support, in combination with face-to-face therapy, or as relapse prevention. Most interventions used a CBT approach, and the remainders were vodcast. The authors found that mHealth interventions had some improvements in symptomatology at post-treatment, although several studies did not have a control group, making it difficult to ascertain the efficacy of this modality.

Pittock and colleagues^38^ reviewed five iCBT studies tested in controlled trials among individuals with bulimic symptoms (i.e., bulimia nervosa or subthreshold bulimia nervosa). One study revealed iCBT to be more efficacious than bibliotherapy and a waitlist in reducing eating disorder behaviors at post-treatment, one study showed iCBT was superior to waitlist controls on self-induced vomiting at post-treatment, and three studies had large effects on binge eating and purging that were maintained at follow up, but no differences compared to controls. iCBT outperformed waitlist treatments in terms of abstinence from binge eating and other behaviors at follow-up. The authors call for more large-scale studies. The authors did not report overall effect sizes.

Melioli et al.^39^ published a meta-analytic review of the efficacy of Internet interventions for reducing eating disorder symptoms and risk factors among individuals with ED symptoms or full-threshold diagnoses. Twenty studies were included that met the review criteria of comparing Internet-based preventive and treatment interventions to no-treatment or minimally-intensive control conditions (e.g., a brochure) using experimental or quasi-experimental designs. Studies that included active treatments as a comparison were excluded from the review. Across all studies, there was an overall summary effect of the interventions for improving eating disorder pathology. Specifically, summary effects were measured for body dissatisfaction (d = 0.28; 95% CI = [0.15, 0.41]; P < 0.001), drive for thinness (d = 0.47; 95% CI = [0.33, 0.60]; P < 0.001), thin ideal internalization (d = 0.36; 95% CI = [0.07, 0.65]; P < 0.05), shape and weight concerns (shape concern: d = 0.35; 95% CI = [0.13, 0.57]; P < 0.05; weight concern: d = 0.25; 95% CI = [0.09, 0.40]; P < 0.05), dietary restriction (d = 0.36; 95% CI = [0.23, 0.49], P < 0.001), bulimic symptoms (d = 0.27; 95% CI = [0.17, 0.37], P < 0.001), purging frequency (d = 0.30; 95% CI = [0.02, 0.57]; P < 0.05), and negative affect (d = 0.32; 95% CI = [0.12, 0.52], P < 0.05). Completer analyses revealed similar findings as intent-to-treat analyses, suggesting that their results were not biased by rates of dropout. The authors concluded that Internet-based interventions successfully decrease eating disorder symptomatology.

In 2015, Schlegl and colleagues^40^ also published a systematic review of technology-based psychological interventions, and they had the goal of extending past prior reviews by including studies that were not limited to RCTs. They evaluated 40 independent studies that evaluated technology-based psychological interventions for individuals with anorexia nervosa or bulimia nervosa, individuals with subthreshold symptoms, and caregivers. They excluded studies of interventions that had been recently reviewed in published manuscripts (e.g., virtual reality, StudentBodies). The authors concluded that guided computer-based interventions were efficacious for improving symptoms of bulimia nervosa (i.e., binge eating, purging, and global eating disorder psychopathology) compared to control conditions among adults, with the need for RCTs among adolescents in this population. However, they noted that poor uptake, dropout, and noncompliance may hinder the validity of their findings. The authors found that only one computer-based intervention had been evaluated for anorexia nervosa, with other interventions focused on relapse prevention in this population.

Loucas and colleagues^41^ evaluated RCTs of e-therapies to prevent and treat eating disorders using the UK's NICE systematic review methodology. E-therapies were defined as interventions delivered by computer, tablet, or mobile phone via the Internet, software, CD-ROM, or an app; comparator conditions were control conditions or an active intervention. E-therapies could include therapist contact, but studies were excluded if the primary mode of delivery was via a therapist (e.g., in-person, face-to-face treatment). Their literature review yielded 20 relevant RCTs but only 4 of which evaluated Internet therapies. At the end of the intervention, when compared with a waitlist control condition, CBT-based e-therapy was associated with small improvements in binge eating, vomiting and/or laxative misuse, and improved rates of cessation of binge eating, vomiting and/or laxative misuse (24% of participants in the intervention group versus 13% in the control group.) However, the few treatment studies involved in the analysis limit meaningful meta-analyses of the data and the authors note that confidence in the effect estimates was low.

Fairburn and Murphy^42^ also reviewed RCTs that evaluated online CBT interventions for eating disorders. They said that of the four trials, comprised of adults with eating disorders characterised by binge eating, engagement was low and cessation rates from binge eating varied from 10% to 37% in intent-to-treat analyses. However, they also said improvements from treatment were maintained at follow up.

Aardoom et al.^43^ identified 21 studies, of which 14 evaluated Internet-based programs. The authors noted that “because of the limited number of studies that compared internet-based treatment to waiting list control conditions, and missing or incomplete data reports…it is hard to reach a reliable conclusion.”

Innovations & Emerging Directions for Eating Disorder Interventions

We identified several articles that used virtual reality for assessing and treatment, and seemed to include iCBT treatments. Clus and colleagues^44^ evaluated 26 studies of which eight were RCTs. They compared interventions using virtual reality techniques (e.g., to address body image or exposure to food stimuli) to another treatment or control. Given the heterogeneity of the studies (e.g., in terms of samples, virtual reality protocols), the authors did not report on the efficacy of the tools or interpret the results of the research. An earlier review, published in 2017, evaluated virtual reality for the assessment and treatment of bulimia nervosa, binge eating disorder, and subthreshold variants^45^. The authors identified 19 clinical studies (i.e., controlled, uncontrolled, or case reports), of which nine were assessment studies and ten were treatment studies, using virtual reality. They concluded that these studies are at an early stage but a promising approach for further study.

Two reviews of smartphone apps in major app stores suggest that apps designed for intervention are available, but few are evidence-based^46^ and their clinical utility is unclear^47^. There also are exciting advances in large-scale implementation efforts to screen individuals with potential eating disorders^48^ and to provide iCBT based preventive and clinical services to populations^49^. Finally, use of social media requires further exploration, given the potential for benefits and harms from this modality among individuals with eating disorders^50, 51^.

Summary

Across studies, results suggest that Internet-based interventions for eating disorders are effective with small to moderate effect sizes. Several themes emerged for future directions for eating disorder research. First, more work is needed to evaluate Internet-based treatment interventions, particularly in comparison to face-to-face treatments. Second, as is relevant for many mental health disorders, opportunities to increase engagement and reduce rates of dropout are warranted. One recent meta-analysis of RCTs involving CBT for eating disorders found the overall dropout rates was about 24% and that dropout from digital CBT interventions was higher than for other delivery modalities (e.g., individual, group)^52^. Third, it will be helpful to continue to identify factors that moderate intervention response in order to establish more precise delivery models and realise the full potential of Internet-based interventions for improving mental health care delivery.

**Discussion**

Across Internet-based intervention studies addressing depression, anxiety, substance abuse, and eating disorders, almost all reviews and meta-analyses found that these interventions can successfully reduce symptoms and are efficacious treatments. Generally, effect sizes for Internet-based interventions treating eating disorders and substance abuse are lower compared to interventions for depression and anxiety. At least for anxiety and depression, therapist supported/guided interventions appear more effective than unguided approaches and comparable to face-to-face. It is not clear why Internet-based interventions for eating disorders and substance abuse seem less effective than for other disorders, but it may reflect the need to address comorbidities and provide longer therapies. More studies are needed to examine such issues.

Dropout continues to be a major issue in Internet-based interventions across disorders. Interventions that provide additional guidance or support can increase adherence; however, this model may not be as scalable as self-help models without guidance or support. Tailoring interventions to reflect an individual’s symptoms, demographic, interests, and technology usage may be a solution to increasing engagement in self-help models and to increase reach of an effective, customised intervention. Additionally, pairing interventions with other apps may be beneficial.

Several of the older reviews and meta-analyses did not include interventions delivered via mobile phone applications, which is an almost ubiquitous technology feature among smartphone users. Future reviews should include more app-based programs as outcome studies become available. Interventions targeting individuals with lower socioeconomic backgrounds and with less access to high-end technology should also be developed and evaluated.

Limitations

There are a few limitations that should be noted. First, we included articles that focused on Internet-based treatment interventions primarily tested among adults; while some reviews and meta-analyses included adolescents and/or preventive interventions, studies focused exclusively on these areas were not included in this review. Second, the research database is strongly dominated by CBT based interventions. Third, as our goal was to summarise the state of the knowledge in this area and provide an overview of the literature, we do not present this as a systematic review or meta-analysis review, nor do we adhere to that format. Future work may benefit from a meta-analysis of the meta-analyses in this area. Finally, as we indicate in our search criteria, we did not include articles that focused on mental health problems in general to maintain scope; however, some articles may have been missed from our overview as a result.

*Conclusion*

Taken together, Internet-based interventions for depression, anxiety, substance abuse, and eating disorders appear efficacious in reducing symptoms. Important areas for future research include generating more evidence for interventions delivered via mobile phone applications, identifying strategies to increase engagement, and targeting digital technologies for various defined populations. The results of this overview strongly support the importance of the ICare program to enhance knowledge related to mobile interventions.

**Acknowledgements**

We want to acknowledge important contributions from Arielle Smith who helped with manuscript preparation.

**Funding**

This research was supported by R01 MH100455, R01 MH115128 and K01 DK116925 from the National Institutes of Health.

*Conflict of Interest:* None declared.

**Key Points**

- Overall, Internet-based interventions for depression, anxiety, substance abuse and eating disorders can successfully reduce symptoms and are efficacious.
- Generally, effect sizes for Internet-based interventions treating eating disorders and substance abuse are lower compared to interventions for depression and anxiety.
- Important areas for future research include generating more evidence for interventions delivered via mobile phone applications, identifying strategies to increase engagement, and targeting digital technologies for various defined populations.

**References**

1. Fairburn CH, Patel V. The impact of digital technology on psychological treatments and their dissemination. Behav Res Ther. 2017;88:19–25. doi: 10.1016/j.brat.2016.08.012

2. Munoz R. The Efficiency Model of Support and the Creation of Digital Apothecaries. Clin Psych: Sci and Prac. 2016;24:46-49. doi: 10.1111/cpsp.12174

3. Oldenburg B, Taylor CB, O’Neil A, et al. Using New Technologies to Improve the Prevention and Management of Chronic Conditions in Populations. Ann Rev Pub Health. 2015;36:483-505. doi: 10.1146/annurev-publhealth-031914-122848

4. [Andrews G](https://www.ncbi.nlm.nih.gov/pubmed/?term=Andrews%20G%5BAuthor%5D&cauthor=true&cauthor_uid=29422409), [Basu A](https://www.ncbi.nlm.nih.gov/pubmed/?term=Basu%20A%5BAuthor%5D&cauthor=true&cauthor_uid=29422409), [Cuijpers P](https://www.ncbi.nlm.nih.gov/pubmed/?term=Cuijpers%20P%5BAuthor%5D&cauthor=true&cauthor_uid=29422409), et al. Computer therapy for the anxiety and depression disorders is effective, acceptable and practical health care: An updated meta-analysis. J Anxiety Disord 2018;55:70-8. doi: 10.1016/j.janxdis.2018.01.001

5. Wright JH, Owen JJ, Richards D, et al. Computer-assisted cognitive-behavior therapy for depression: A systematic review and meta-analysis. J Clin Psychiatry. 2019;80(2)18r12188. doi: 10.4088/JCP.18r12188

6. [Josephine K](https://www.ncbi.nlm.nih.gov/pubmed/?term=Josephine%20K%5BAuthor%5D&cauthor=true&cauthor_uid=28715726), [Josefine L](https://www.ncbi.nlm.nih.gov/pubmed/?term=Josefine%20L%5BAuthor%5D&cauthor=true&cauthor_uid=28715726), [Philipp D](https://www.ncbi.nlm.nih.gov/pubmed/?term=Philipp%20D%5BAuthor%5D&cauthor=true&cauthor_uid=28715726), [David E](https://www.ncbi.nlm.nih.gov/pubmed/?term=David%20E%5BAuthor%5D&cauthor=true&cauthor_uid=28715726), [Harald B](https://www.ncbi.nlm.nih.gov/pubmed/?term=Harald%20B%5BAuthor%5D&cauthor=true&cauthor_uid=28715726). Internet- and mobile-based depression interventions for people with diagnosed depression: A systematic review and meta-analysis. J Affect Disord 2017;223:28-40. doi: 10.1016/j.jad.2017.07.021. (The correct authors are: Königbauer, J., Letsch, J., Doebler, P., Ebert, D. Baumeister, H.)

7. Karyotaki E, Riper H, Twisk J, et al. Efficacy of Self-guided Internet-Based Cognitive Behavioral Therapy in the Treatment of Depressive Symptoms: A Meta-analysis of Individual Participant Data. JAMA Psych. 2017;74(4):351-359. doi: 10.1001/jamapsychiatry.2017.0044

8. Karyotaki E, Ebert DD, Donkin L, et al. Do guided internet-based interventions result in clinically relevant changes for patients with depression? An individual participant data meta-analysis. Clin Psychol Rev 2018;63:80-92. doi: 10.1016/j.cpr.2018.06.007

9. Wahle F, Bollhalder L, Kowatsch T, Fleisch E. Toward the Design of Evidence-Based Mental Health Information Systems for People With Depression: A Systematic Literature Review and Meta-Analysis. J Med Internet Res. 2017 May 31;19(5):e191. doi: 10.2196/jmir.7381

10. Twomey C, O'Reilly G, Meyer B. Effectiveness of an individually-tailored computerised CBT programme (Deprexis) for depression: A meta-analysis. Psychiatry Research 2017;256:371-377. doi: 10.1016/j.psychres.2017.06.081

11. Arnberg FK, Linton SJ, Hultcrantz M, Heintz E, Jonsson U. Internet-Delivered Psychological Treatments for Mood and Anxiety Disorders: A Systematic Review of Their Efficacy, Safety, and Cost-Effectiveness. PLoS One 2014;9(5):1-13. doi: 10.1371/journal.pone.0098118

12. Thase ME, Wright JH, Eells TD, et al. [Improving the Efficiency of Psychotherapy for Depression: Computer-Assisted Versus Standard CBT.](https://www.ncbi.nlm.nih.gov/pubmed/28969439) Am J Psychiatry 2018;175(3):242-250. doi: 10.1176/appi.ajp.2017.17010089

13. Klein JP, Gamon C, Späth C, et al. [Does recruitment source moderate treatment effectiveness? A subgroup analysis from the EVIDENT study, a randomised controlled trial of an Internet intervention for depressive symptoms.](https://www.ncbi.nlm.nih.gov/pubmed/28710212) BMJ Open 2017;7(7):e015391. doi: 10.1136/bmjopen-2016-015391

14. Păsărelu CR, Andersson G, Bergman Nordgren L, Dobrean A. [Internet-delivered transdiagnostic and tailored cognitive behavioral therapy for anxiety and depression: a systematic review and meta-analysis of randomized controlled trials.](https://www.ncbi.nlm.nih.gov/pubmed/27712544) Cogn Behav Ther. 2017;46(1):1-28. doi: 10.1080/16506073.2016.1231219

15. Richards D, Richardson T. Computer-based psychological treatments for depression: A systematic review and meta-analysis. Clin Psychol Rev 2012;32(4):329-342. doi: 10.1016/j.cpr.2012.02.004

16. [Hedman E](https://www.ncbi.nlm.nih.gov/pubmed/?term=Hedman%20E%5BAuthor%5D&cauthor=true&cauthor_uid=23252357), [Ljótsson B](https://www.ncbi.nlm.nih.gov/pubmed/?term=Lj%C3%B3tsson%20B%5BAuthor%5D&cauthor=true&cauthor_uid=23252357), [Lindefors N](https://www.ncbi.nlm.nih.gov/pubmed/?term=Lindefors%20N%5BAuthor%5D&cauthor=true&cauthor_uid=23252357). Cognitive behavior therapy via the Internet: a systematic review of applications, clinical efficacy and cost-effectiveness. [Expert Rev Pharmacoecon Outcomes Res.](https://www.ncbi.nlm.nih.gov/pubmed/23252357) 2012 Dec;12(6):745-64. doi: 10.1586/erp.12.67

17. [Carlbring P](https://www.ncbi.nlm.nih.gov/pubmed/?term=Carlbring%20P%5BAuthor%5D&cauthor=true&cauthor_uid=29215315), [Andersson G](https://www.ncbi.nlm.nih.gov/pubmed/?term=Andersson%20G%5BAuthor%5D&cauthor=true&cauthor_uid=29215315), [Cuijpers P](https://www.ncbi.nlm.nih.gov/pubmed/?term=Cuijpers%20P%5BAuthor%5D&cauthor=true&cauthor_uid=29215315), [Riper H](https://www.ncbi.nlm.nih.gov/pubmed/?term=Riper%20H%5BAuthor%5D&cauthor=true&cauthor_uid=29215315), [Hedman-Lagerlöf E](https://www.ncbi.nlm.nih.gov/pubmed/?term=Hedman-Lagerl%C3%B6f%20E%5BAuthor%5D&cauthor=true&cauthor_uid=29215315). Internet-based vs. face-to-face cognitive behavior therapy for psychiatric and somatic disorders: an updated systematic review and meta-analysis. Cogn Behav Ther 2018;47(1):1-18. doi: 10.1080/16506073.2017.1401115

18. Karyotaki E, Kemmeren L, Riper H, et al. Is self-guided internet-based cognitive behavioural therapy (iCBT) harmful? An individual participant data meta-analysis. Psychol Med 2018;48(15):2456-2466. doi: 10.1017/S0033291718000648

19. Roepke A, Jaffee S, Riffle O, McGonigal J, Broome R, Maxwell B. Randomized Controlled Trial of SuperBetter, a Smartphone-Based/Internet-Based Self-Help Tool to Reduce Depressive Symptoms. Games Health J 2015;4(3):235-46. doi: 10.1089/g4h.2014.0046

20. Lau H, Smit J, Fleming T, Riper H. [Serious Games for Mental Health: Are They Accessible, Feasible, and Effective? A Systematic Review and Meta-analysis.](https://www.ncbi.nlm.nih.gov/pubmed/28149281) Front Psychiatry 2017;7:209. doi: 10.3389/fpsyt.2016.00209

21. Aguilera A, Bruehlman-Senecal E, Demasi O, Avila P. [Automated Text Messaging as an Adjunct to Cognitive Behavioral Therapy for Depression: A Clinical Trial.](https://www.ncbi.nlm.nih.gov/pubmed/28483742) J Med Internet Res 2017;19(5):e148. doi: 10.2196/jmir.6914

22. [Berrouiguet S](https://www.ncbi.nlm.nih.gov/pubmed/?term=Berrouiguet%20S%5BAuthor%5D&cauthor=true&cauthor_uid=27287668), [Baca-García E](https://www.ncbi.nlm.nih.gov/pubmed/?term=Baca-Garc%C3%ADa%20E%5BAuthor%5D&cauthor=true&cauthor_uid=27287668), [Brandt S](https://www.ncbi.nlm.nih.gov/pubmed/?term=Brandt%20S%5BAuthor%5D&cauthor=true&cauthor_uid=27287668), [Walter M](https://www.ncbi.nlm.nih.gov/pubmed/?term=Walter%20M%5BAuthor%5D&cauthor=true&cauthor_uid=27287668), [Courtet P](https://www.ncbi.nlm.nih.gov/pubmed/?term=Courtet%20P%5BAuthor%5D&cauthor=true&cauthor_uid=27287668). Fundamentals for Future Mobile-Health (mHealth): A Systematic Review of Mobile Phone and Web-Based Text Messaging in Mental Health. J Med Internet Res 2016;18(6):e135. doi: 10.2196/jmir.5066

23. Schueller S, Aguilera A, Mohr D. Ecological momentary interventions for depression and anxiety. Depress Anxiety 2017;34(6):540-545. doi: 10.1002/da.22649

24. Provoost S, Lau HM, Ruwaard J, Riper H. [Embodied Conversational Agents in Clinical Psychology: A Scoping Review.](https://www.ncbi.nlm.nih.gov/pubmed/28487267) J Med Internet Res 2017;19(5):e151. doi: 10.2196/jmir.6553

25. Mohr DC, Tomasino KN, Lattie EG, et al. [IntelliCare: An Eclectic, Skills-Based App Suite for the Treatment of Depression and Anxiety.](https://www.ncbi.nlm.nih.gov/pubmed/28057609) J Med Internet Res 2017;19(1):e10. doi: 10.2196/jmir.6645

26. Taylor CB, Ruzek, J, Fitzsimmons-Craft, Graham AK, Balantekin K. A systematic digital approach to implementation and dissemination of eating disorders interventions. mHealth 2018;4(25):1-13. doi: 10.21037/mhealth.2018.05.06

27. [Olthuis JV](https://www.ncbi.nlm.nih.gov/pubmed/?term=Olthuis%20JV%5BAuthor%5D&cauthor=true&cauthor_uid=26968204), [Watt MC](https://www.ncbi.nlm.nih.gov/pubmed/?term=Watt%20MC%5BAuthor%5D&cauthor=true&cauthor_uid=26968204), [Bailey K](https://www.ncbi.nlm.nih.gov/pubmed/?term=Bailey%20K%5BAuthor%5D&cauthor=true&cauthor_uid=26968204), [Hayden JA](https://www.ncbi.nlm.nih.gov/pubmed/?term=Hayden%20JA%5BAuthor%5D&cauthor=true&cauthor_uid=26968204), [Stewart SH](https://www.ncbi.nlm.nih.gov/pubmed/?term=Stewart%20SH%5BAuthor%5D&cauthor=true&cauthor_uid=26968204). Therapist-supported Internet cognitive behavioural therapy for anxiety disorders in adults. Cochrane Database Syst Rev 2016;3:CD011565. doi: 10.1002/14651858.CD011565

28. [Peñate W](https://www.ncbi.nlm.nih.gov/pubmed/?term=Pe%C3%B1ate%20W%5BAuthor%5D&cauthor=true&cauthor_uid=26026188), [Fumero A](https://www.ncbi.nlm.nih.gov/pubmed/?term=Fumero%20A%5BAuthor%5D&cauthor=true&cauthor_uid=26026188). A meta-review of Internet computer-based psychological treatments for anxiety disorders. J Telemed Telecare 2016;22(1):3-11. doi: 10.1177/1357633X15586491

29. [Kampmann IL](https://www.ncbi.nlm.nih.gov/pubmed/?term=Kampmann%20IL%5BAuthor%5D&cauthor=true&cauthor_uid=27376634), [Emmelkamp PM](https://www.ncbi.nlm.nih.gov/pubmed/?term=Emmelkamp%20PM%5BAuthor%5D&cauthor=true&cauthor_uid=27376634), [Morina N](https://www.ncbi.nlm.nih.gov/pubmed/?term=Morina%20N%5BAuthor%5D&cauthor=true&cauthor_uid=27376634). Meta-analysis of technology-assisted interventions for social anxiety disorder. J Anxiety Disord 2016;42:71-84. doi: 10.1016/j.janxdis.2016.06.007

30. Sijbrandij M, Kunovski I, Cuijpers P. Effectiveness of Internet-delivered cognitive behavioral therapy for posttraumatic stress disorder: a systematic review and meta-analysis. Depress Anxiety 2016;33(9):783–791. doi: 10.1002/da.22533

31. Kaner EFS, Beyer FR, Garnett C, et al. Personalised digital interventions for reducing hazardous and harmful alcohol consumption in community-dwelling populations. Cochrane Database of Systematic Reviews 2017; 9:CD011479. doi: 10.1002/14651858.CD011479.pub2

32. [Prosser T](https://www.ncbi.nlm.nih.gov/pubmed/?term=Prosser%20T%5BAuthor%5D&cauthor=true&cauthor_uid=29452058), [Gee KA](https://www.ncbi.nlm.nih.gov/pubmed/?term=Gee%20KA%5BAuthor%5D&cauthor=true&cauthor_uid=29452058), [Jones F](https://www.ncbi.nlm.nih.gov/pubmed/?term=Jones%20F%5BAuthor%5D&cauthor=true&cauthor_uid=29452058). A meta-analysis of effectiveness of E-interventions to reduce alcohol consumption in college and university students. [J Am Coll Health.](https://www.ncbi.nlm.nih.gov/pubmed/?term=Prosser+T%2C+Gee+KA) 2018;66(4):292-301. doi: 10.1080/07448481.2018.1440579

33. Black N, Mullan B, Sharpe L. Computer-delivered interventions for reducing alcohol consumption: meta-analysis and meta-regression using behaviour change techniques and theory. Health Psychol Rev 2016;7199(April):1–33. doi: 10.1080/17437199.2016.1168268

34. Riper H, Blankers M, Hadiwijaya H, et al. Effectiveness of Guided and Unguided Low-Intensity Internet Interventions for Adult Alcohol Misuse: PLoS ONE 2014;9(6):e99912. doi: 10.1371/journal.pone.0099912

35. [Boumparis N](https://www.ncbi.nlm.nih.gov/pubmed/?term=Boumparis%20N%5BAuthor%5D&cauthor=true&cauthor_uid=28295758), [Karyotaki E](https://www.ncbi.nlm.nih.gov/pubmed/?term=Karyotaki%20E%5BAuthor%5D&cauthor=true&cauthor_uid=28295758), [Schaub MP](https://www.ncbi.nlm.nih.gov/pubmed/?term=Schaub%20MP%5BAuthor%5D&cauthor=true&cauthor_uid=28295758), [Cuijpers P](https://www.ncbi.nlm.nih.gov/pubmed/?term=Cuijpers%20P%5BAuthor%5D&cauthor=true&cauthor_uid=28295758), [Riper H](https://www.ncbi.nlm.nih.gov/pubmed/?term=Riper%20H%5BAuthor%5D&cauthor=true&cauthor_uid=28295758). Internet interventions for adult illicit substance users: a meta-analysis. Addiction 2017;112(9):1521-1532. doi: 10.1111/add.13819

36. Tait RJ, Spijkerman R, Riper H. Internet and computer based interventions for cannabis use: A meta-analysis. Drug and Alcohol Dependence 2013;133(2):295-304. doi: 10.1016/j.drugalcdep.2013.05.012

37. Anastasiadou D, Folkvord F, Lupianez-Villanueva F. A systematic review of mHealth interventions for the support of eating disorders. Eur Eat Disord Rev. 2018;26(5):394-416. doi: 10.1002/erv.2609

38. Pittock A, Hodges L, Lawrie SM. The effectiveness of internet-delivered cognitive behavioural therapy for those with bulimic symptoms: a systematic review. BMC Res Notes 2018;11(1):748. doi: 10.1186/s13104-018-3843-2

39. Melioli T, Bauer S, Franko DL, et al. Reducing eating disorder symptoms and risk factors using the internet: A meta-analytic review. Int J Eat Disord 2016;49(1):19-31. doi: 10.1002/eat.22477

40. Schlegl S, Burger C, Schmidt L, Herbst N, Voderholzer U. The Potential of Technology-Based Psychological Interventions for Anorexia and Bulimia Nervosa: A Systematic Review and Recommendations for Future Research. J Med Internet Res 2015;17(3):e85. doi: 10.2196/jmir.3554

41. Loucas CE, Fairburn CG, Whittington C, Pennant ME, Stockton S, Kendall T. E-therapy in the treatment and prevention of eating disorders: A systematic review and meta-analysis. Behav Res Ther 2014;63:122-131. doi: 10.1016/j.brat.2014.09.011

42. Fairburn CG, Murphy R. Treating eating disorders using the Internet. Curr Opin Psychiatry. 2015;28(6):461-7. doi: 10.1097/YCO.0000000000000195

43. Aardoom JJ, Dingemans AE, Spinhoven P, Van Furth EF. Treating eating disorders over the internet: A systematic review and future research directions. Int J Eat Disord 2013;46(6):539-552. doi: 10.1002/eat.22135

44. Clus D, Larsen ME, Lemey C, Berrouiguet S. The Use of Virtual Reality in Patients with Eating Disorders: Systematic Review. J Med Internet Res. 2018;20(4):e157. doi: 10.2196/jmir.7898

45. de Carvalho MR, Dias TRS, Duchesne M, Nardi AE, Appolinario JC. Virtual Reality as a Promising Strategy in the Assessment and Treatment of Bulimia Nervosa and Binge Eating Disorder: A Systematic Review. Behav Sci (Basel). 2017;7(3). doi: 10.3390/bs7030043

46. Juarascio AS, Manasse SM, Goldstein SP, Forman EM, Butryn ML. Review of Smartphone Applications for the Treatment of Eating Disorders. Eur Eat Disord Rev 2015;23(1):1-11. doi: 10.1002/erv.2327

47. Fairburn CG, Rothwell ER. Apps and eating disorders: A systematic clinical appraisal. Int J Eat Disord. 2015;48(7):1038-46. doi: 10.1002/eat.22398

48. Fitzsimmons-Craft AE, Balantekin KN, Graham AK, et al. Results of disseminating an online screen for eating disorders across the U.S.: Reach, respondent characteristics, and unmet treatment need. Int J of Eat Disord, 2019;52(6):721-729. doi: 10.1002/eat.23043

49. Fitzsimmons-Craft EE, Firebaugh ML, Graham AK, et al. [State-wide university implementation of an online platform for eating disorders screening and intervention](https://www.ncbi.nlm.nih.gov/pubmed/30407047). Psychol Serv. 2019;16(2):239-249. doi: 10.1037/ser0000264

50. Saffran K, Fitzsimmons-Craft EE, Kass AE, Wilfley DE, Taylor CB, Trockel M. Facebook usage among those who have received treatment for an eating disorder in a group setting. Int J Eat Disord 2016;49(8):764-77. doi: 10.1002/eat.22567

51. Mabe AG, Forney KJ, Keel PK. Do you "like" my photo? Facebook use maintains eating disorder risk. Int J Eat Disord 2014;47(5):516-23. doi: 10.1002/eat.22254

52. [Linardon J](https://www.ncbi.nlm.nih.gov/pubmed/?term=Linardon%20J%5BAuthor%5D&cauthor=true&cauthor_uid=29493805), [Hindle A](https://www.ncbi.nlm.nih.gov/pubmed/?term=Hindle%20A%5BAuthor%5D&cauthor=true&cauthor_uid=29493805), [Brennan L](https://www.ncbi.nlm.nih.gov/pubmed/?term=Brennan%20L%5BAuthor%5D&cauthor=true&cauthor_uid=29493805). Dropout from cognitive-behavioral therapy for eating disorders: A meta-analysis of randomized, controlled trials. Int J Eat Disord 2018;51(5):381-91. doi: 10.1002/eat.22850
