# Supplementary material for: Current state of scientific evidence on Internet-based interventions for the treatment of depression, anxiety, eating disorders and substance abuse: an overview of systematic reviews and meta-analyses
Source: Eur J Public Health. 2020 Sep 11;31(Suppl 1):i3–i10. doi: 10.1093/eurpub/ckz208 (PMC8495688; doi:10.1093/eurpub/ckz208)
Supplement: ckz208_Supplementary_Data [file ckz208_supplementary_data.zip › ckz208-suppl_data/Supplementary TableFinal10.1.19.docx]

**Supplementary Table 1.** Specifics of studies reviewed.

| **Study** | Guidelines | S | N | Interventions | Comparators | Outcome Measures | Follow-up  (months) | R | Effect size range  (RX versus control) | A |
| --- | --- | --- | --- | --- | --- | --- | --- | --- | --- | --- |
| **DEPRESSION** | | | | | | | | | | |
| Andrews et al. (1) | PRISMA | 32 | 5642 | CBT | Support, WLC, Information,  Discussion group,  AP | BDI, BDI-II, CES-D, PHQ-9, Ham-D | 1-5: 9  6-8: 11  9+: 5  Unclear: 7 | Y | Post Hedges’ g= 0.67, 95% CI [0.51,  -0.81) | Y |
| Arnberg et al. (2) | None | 8 | 958 | CBT (7/8), PDT | AC, TAU, WLC | BDI, non-depression related measures may have been used in one study | Not clear | Y | Z = 6.75; 95% CI =  [-0.42, -0.14]; P < 0.001 | N |
| Hedman et al. (3) | None | 20 | 4776 | CBT | “another treatment” | BDI, BDI II,  CES-D, K10,  PHQ9 | Not Clear | N | d = 0.94; 95% CI = [0.77, 1.11]). | N |
| Josephine et al. (4) | PROS-PERO  CRD42016039679 | 19 | 1650 | CBT (11/19), CBM,  Physical activity, Psychodynamic,  PST, ‘Spiritually informed’ | Group CBtT,  WLC, 5 had no comparator | BDI, BDI-II, CES-D, Ham-D, HDRS, MADRS, PHQ-9 | None: 2  1-5: 7  6-8: 6  9+: 4 | N | Within group SMD range = -2.24 to  -0.64) and from pre-treatment to follow-up (SMD range = -3.07 to 0.93). | Y |
| Karyotaki et al. (5) | PRISMA | 13 | 3876* | Unguided iCBT | AP, no treatment, TAU, WLC | BAI I or II, CEDS, PHQ-9, others standardized by Z score | Unclear:  Apparently Post-Rx  only | Y | Hedges’ g  = 0.27 (95% CI = [0.17 to 0.37]; P < 0.001). | Y |
| Karyotaki et al. (6) | PRISMA | 24 | 4889* | Guided iCBT (17/24), CBM, PST | Discussion, Education, Self-help book,  WLC, TAU | BAI I or II, CEDS, PHQ-9, others standardized by Z score | 5 weeks to 3 months | Y | Odds Ratio (OR) = 2.49; 95% CI = [2.17, 2.85]) and remission rates compared to controls (OR = 2.41; 95% CI = [2.07, 2.79]). | N |
| Richards & Richardsons (7) | None | 19 | 3291 | CBT(17/19),GCBT, email, PST | Self-help,  Discussion, TAU, WL | BDI, CES-D, GDS, MDRAS, HDRS | Not clear | Y | d = 0.56; 95% CI = [−0.71, −0.41]; Z = 7.48; P *<* 0.001 | N |
| Twomey et al. (8) | None | 8 | 2402 | CBT (Only included results from the Deprexis program) | TAU, WLC | BDI-I, BDI-II, PHQ-9, QIDS | 8 to 12 weeks | Y | g = 0.54; 95% CI = [0.39, 0.69] | N |
| Wahle et al. (9) | PRISMA | 45** | 4519 | CBT (guided and unguided, blended 34/45), PDT, Supportive messages, | ACT,  Group CBT, Discussion group, Web discussion,  depression rx, TAU, WLC, other | BDI, 1A or II, PHQ, PHQ 2 or PHQ9 | 6 to 14 weeks | Y | SMD =  –0.58; 95% CI = [–0.71,  –0.45]; P < 0.001 | Y |
| Wright et al. (10) | PRISMA | 40 | 7198 | CBT | Support, WLC, information,  Discussion group,  AP, TAU | BDI, BDI-II, CES-D, CIS-R, EPDS, Ham-D, HDRS, KPDS, MADRS, MINI, PHQ-9, QIDS, SCID | Unclear.  Authors report range as 1-6 | Y | The random effects weighted mean effect size for iCBT versus controls at posttreatment was g = 0.50 (SE = 0.06; 95% CI = [0.39, 0.61]; P < 0.001) | Y |
| **ANXIETY –SYMPTOMS AND DISORDERS (COMBINED)** | | | | | | | | | | |
| Hedman et al. (3) | None | 6 | 608 | CBT; “spiritual internet treatment” | “another treatment” | BAI, HAI, DASS-21, PSS | UC/M | N | d = 1.07; 95% CI = [0.75, 1.39] | N |
| Olthuis et al.  (11) | PRISMA | 30 | 2181 | BT, CBT, CT, +/- support, face-to-face | AC, IC, D, WLC | Clinically important change | UC/M | Y | RR = 4.18 (95% [CI 2.42 to 7.22] for clinically important anxiety; Disorder-specific improvement: (22 studies, n = 1573, SMD = -1.12, 95% CI [-1.39 to -0.85] and general anxiety symptoms at post-treatment (14 studies, n = 1004, SMD= -0.79, 95% CI [-1.10 to -0.48) favored therapist-supported ICBT | Y |
| **ANXIETY – PANIC DISORDER, AGORAPHOBIA** | | | | | | | | | | |
| Andrews et al.  (1) | PRISMA | 12 | 555 | CBT +/- support | IC, stress modules,  WLC | BSQ, PDSS-SR | 1-5: 5  6-8: 2  9+: 2  UC/M: 3 | Y | Post Hedges’ g= 1.31, 95% CI [0.85, 1.76]; P = 0.00) | Y |
| Arnberg et al. (2) | None | 9 | No meta-analysis was undertaken because of the heterogeneity in outcome measures and low quality of evidence | | | | | | | |
| Hedman et al. (3) | None | 9 | 407 | CBT | “another treatment” | BSQ, PDSS-SR | UC/M | N | d = 1.42; 95% CI = [0.86, 1.99] | N |
| **ANXIETY – SOCIAL ANXIETY DISORDER** | | | | | | | | | | |
| Andrews et al. (1) | PRISMA | 11 | 950 | CBT | WLC | BFNE, LSAS-SR, SIAS, SPS | 1-5: 0  6-8: 3  9+: 5  UC/M: 3 | Y | Post Hedges’ g= 0.92, 95% CI [0.51,  -0.81]; P = 0.05) | Y |
| Arnberg et al. (2) | None | 13 | NR | CBT | D, B, Sham Rx, WLC | SIAS | SIAS | Y | d = 0.85 (95% CI = [0.66, 1.05]; P < 0.001 | N |
| Hedman et al. (3) | None | 16 | 1448 | CBT | “another treatment” | FPSQ, LSAS, SPS, other | UC/M | N | d = 1.13; 95% CI = [0.99, 1.28] | N |
| Kampmann et al. (12) | None | 37 | 2991 | CBT, ABM, VRET | B, D, IntRx, WLC, Other | FPSQ, LSAS, Placebo training (for VRET), other | 1 to 12 months | Y | g = 0.84; 95% CI = [0.72, 0.97]; P < 0.001 (c/t passive controls); g = 0.38; 95% CI = [0.13, 0.62]; P < 0.01 (c/t active controls) | N |
| **ANXIETY – GENERALIZED ANXIETY DISORDER** | | | | | | | | | | |
| Andrews et al.  (1) | PRISMA | 9 | 1103 | CBT +/-  support | WLC | GAD-7, PSWQ | 1-5: 9  6-8: 11  9+: 5  UC/M: 7 | Y | Post Hedges’ g= 0.70, 95% CI [0.39,1.01]; P = 0.00 | Y |
| Arnberg et al. (2) | None | 4 | NR | CBT | WLC | NR | UC/M | Y | Z = 4.21 (P<.0001); SMD 0.94 [0.45, 1.23] | N |
| Hedman et al. (3) | None | 2 | 145 | CBT | “another treatment” | PSWQ | UC/M | N | D = 1.12; 95% CI = [0.61. 1.62] | N |
| **ANXIETY – PTSD (POST TRAUMATIC STRESS DISORDER)** | | | | | | | | | | |
| Hedman et al. (3) | None | 6 | 148 | CBT | “another treatment” | IES-R, PTSD Symptom Scale, PCL | UC/M | N | d = 1.23; 95% CI = [0.83, 1.63] | N |
| Sijbrandij et al (13) | None | 12 | 1139 | CBT | Support, TAU, WLC | BSI, IESPDS, MPSSPCL, PTS, | UC | Y | g = 0.71; 95% CI = [0.49, 0.93]; P < 0.001 (c/t passive controls); g = 0.28; 95% CI = [0.00, 0.56]; P = 0.05 (c/t other interventions | N |
| **SUBSTANCE ABUSE** | | | | | | | | | | |
| Black et al. (14) | None | 93 | NR | Unguided computer-delivered interventions (employed MI, social norms, health behavior model, social cognitive theory, planned behavior, and transtheoretical models) | Assessment-only or assessment plus intervention  for unrelated health behavior | Total quantity of alcohol consumed; average quantity of  alcohol consumed per drinking occasion; highest quantity of alcohol consumed on one occasion; frequency  of heavy episodic drinking; frequency of alcohol consumption | Unclear | N | Effect sizes ranged from d= 0.07, 95% CI = [0.04, 0.10] to d = 0.15, 95% CI [0.11, 0.18] | N |
| Boumparis et al. (15) | None | 17 | 2836 | Community Reinforcement Approach, CBT, CM, MI, Brief Intervention, Medication | TAU, WLC, Add-on Intervention, MI, Brief Intervention | Toxicology screens, mean maximum  number of days or weeks abstinent throughout  treatment,  consumption within previous weeks or months, self-report measures including DUDIT and ASSIST | 6 to 12 months | Y | g = 0.31; 95% CI = [0.23, 0.39] | Y |
| Kaner et al. (16) | Cochrane | 57 | 34390 | Computer and mobile interventions | No intervention, assessment only, TAU,  or face-to-face interventions for reducing hazardous or harmful alcohol consumption | Drinks per week, drinking frequency, intensity of drinking | 1 to 12 months | Y | 95% CI = [15.4 to 30.3] | N |
| Prosser et al. (17) | None | 23 | 7614 | Personalized feedback, MI, skills training, protective behavioral strategies, education | Assessment only | Drinks per week | 6 to 12 months | Y | Z = 4.80,  SMD = -0.15, CI 95%  [-0.21, -0.09] | N |
| Riper et al. (18) | None | 16 | 5612 | Guided and unguided CBT, MI, PNF, health education, behavioral self-control training | WLC, alcohol or health brochure, assessment only | Mean level of  alcohol consumption, drinking within low-risk guidelines for alcohol consumption | 6 to 12 months | Y | g = 0.20, 95% CI= [0.13, 0.27] | Y |
| Tait et al. (19) | None | 10 | 4125 | CBT, MI, PCT, Universal/Family Prevention, Personalized Feedback | WL or Active (e.g., TAU, information) | Frequency and amount of cannabis used | NC | Y | g = 0.16; 95% CI = [0.09, 0.22] | N |
| **EATING DISORDERS** | | | | | | | | | | |
| Aardoom et al. (20) | Cochrane | 14^†^ | 1405 | Internet-based programs | Waiting list control, unguided self-help, guided biblotherapy | EDE global, EDE-Q global, binge eating, vomiting, BITE | No follow up to 18 months | Y | Effect sizes ranged from d=0.39 to 1.32 (post) and: d=0.06 to 0.99 (follow-up) | Y |
| Anastasiadou et al. (21) | PRISMA | 15 | 58501 | CBT, vodcast | CBTgst, TAU, None, music, other vodcasts, internet-based weight management | Objective binge episodes; attentional bias to food, smoothie consumption | <1 month to 9 months | N | Between-group effect sizes ranged from 0.4 to 0.51 | Y |
| Fairburn & Murphy (22) | Not reported | 4 | 410 | Guided eTherapy (CBT) | Delayed treatment or bibliotherapy | Cessation from binge eating and purging | 3 to 12 months | N | Effect sizes not reported; Cessation at end-of-treatment = 10% to 37%; follow-up = 8% to 43% | Y |
| Loucas et al. (23) | PRISMA | 20 | 2491 | E-therapies designed to prevent or treat eating disorders | WLC, bibliotherapy, TAU, other control conditions | Eating disorder psychopathology | There were FUs, but not specified | Y | For prevention, CBT-based e-intervention associated with small reductions in eating disorder psychopathology (SMDs  -0.30 to  -0.43); for treatment and relapse prevention, various e-therapies showed some beneficial effects but evidence usually came from a single study (SMDs -0.33 to -0.56) | N |
| Melioli et al. (24) | PRISMA | 20 | 1,918 | Internet-based preventive and treatment interventions | WLC, TAU, brochure | Most commonly used:  EDE-Q, EDI, BSQ, WCS  Others:  BDI-II, BDISF, BCS, TFEQ, EAT-40, BITE, BULIT-R, DEBQ-R, PACS, SATAQ | Follow-up not reported on; length of interventions unclear | Y | Body dissatisfaction (d = 0.28); drive for thinness (d = 0.47); thin- ideal internalization (d = 0.36); shape and weight concerns (ds = 0.25 to 0.42); dietary restriction (d = 0.36); bulimic symptoms (d = 0.27); purging frequency (d = 0.30); negative affect (d = 0.32) | N |
| Pittock et al. (25) | PRISMA, Cochrane | 5 | 594 | iCBT | WLC, bibliotherapy, face-to-face CBT | Binge eating and purging; EDE | 4 to 18 months | Y | Binge eating & purging = not sign. in all but 1 study;  EDE d = 1.23 (CI = 1.74 to 0.70) at post in 1 study | N |
| Schlegl et al. (26) | PRISMA | 40 | 3,646 | Technology-based psychological interventions | Various; uncontrolled studies also included | Eating disorder psychopathology | 2 months to 2 years | Y | Significant between-group effect sizes at post ranging from  -0.50 to -2.06; sign. Between-group effect sizes at follow-up ranging from  -0.43 to -0.97 | Y |

Abbreviations: A = adherence assessed, AC = attention control, ACT = Acceptance and Commitment therapy, ASSIST = Alcohol, Smoking, and Substance Involvement Screening Test, BDI = Beck Depression Inventory, BDI-II = Beck Depression Inventory-2nd Edition, BDISF = Beck Depression Inventory short form, BITE = Bulimic Investigatory Test Edinburgh, BULIT-R = Bulimia Test-Revised, CBT = cognitive-behaviour therapy, CBTgsh = cognitive behaviour therapy guided self-help, CCBT = computer-assisted cognitive-behaviour therapy, CBM = cognitive bias modification, CES-D = Center for Epidemiologic Studies Depression Scale, CIS-R = Clinical Interview Schedule-Revised, CM = Contingency Management, DEBQ-R = Dutch Eating Behavior Questionnaire-Restraint Subscale, DUDIT = Drug Use Disorders Identification Test, EAT-40 = Eating Attitudes Test, EDE = Eating Disorders Examination; EDE-Q = Eating Disorders Examination-Questionnaire; EDI = Eating Disorders Inventory; EPDS = Edinburgh Postnatal Depression Scale, GDS, Goldberg Depression Scale, Ham-D, HDRS = Hamilton Depression Rating Scale, KPDS = Kessler Psychological Distress Scale (K10), MADRS = Montgomery-Asberg Depression Rating Scale, MDD = major depressive disorder, MI = Motivational Interviewing, MINI = Mini-International Neuropsychiatric Interview, NR = not reported, PACS = Physical Appearance Comparison Scale, PCT = person-centered therapy, PDT = psychodynamic therapy, PHQ-9 = 9-item Patient Health Questionnaire,  PNF = personalized normative feedback, PST = problem-solving therapy, QIDS = Quick Inventory of Depressive Symptomatology, R= Risk Bias Reported, S = Studies, SATAQ = Sociocultural Attitudes Toward Appearance Questionnaire, SCID = Structured Clinical Interview for DSM-IV-TR Axis I Disorders–Patient Edition, TAU = treatment as usual. TCBT = transdiagnostic CBT, TFEQ = Three-Factor Eating Questionnaire, WCS = Weight Concerns Scale

*Data was analyzed at a participant level from data obtained from the 13 studies

**Includes 7 studies with subjects with depression and comorbid anxiety

^†^ Includes only studies of Internet-based treatments

**References**

1. [Andrews G](https://www.ncbi.nlm.nih.gov/pubmed/?term=Andrews%20G%5BAuthor%5D&cauthor=true&cauthor_uid=29422409), [Basu A](https://www.ncbi.nlm.nih.gov/pubmed/?term=Basu%20A%5BAuthor%5D&cauthor=true&cauthor_uid=29422409), [Cuijpers P](https://www.ncbi.nlm.nih.gov/pubmed/?term=Cuijpers%20P%5BAuthor%5D&cauthor=true&cauthor_uid=29422409), et al. Computer therapy for the anxiety and depression disorders is effective, acceptable and practical health care: An updated meta-analysis. J Anxiety Disord 2018;55:70-8. doi: 10.1016/j.janxdis.2018.01.001
2. Arnberg FK, Linton SJ, Hultcrantz M, Heintz E, Jonsson U. Internet-delivered psychological treatments for mood and anxiety disorders: A systematic review of their efficacy, safety, and cost-effectiveness. PLoS One 2014;9(5):1-13. doi: 10.1371/journal.pone.0098118.
3. [Hedman E](https://www.ncbi.nlm.nih.gov/pubmed/?term=Hedman%20E%5BAuthor%5D&cauthor=true&cauthor_uid=23252357), [Ljótsson B](https://www.ncbi.nlm.nih.gov/pubmed/?term=Lj%C3%B3tsson%20B%5BAuthor%5D&cauthor=true&cauthor_uid=23252357), [Lindefors N](https://www.ncbi.nlm.nih.gov/pubmed/?term=Lindefors%20N%5BAuthor%5D&cauthor=true&cauthor_uid=23252357). Cognitive behavior therapy via the Internet: a systematic review of applications, clinical efficacy and cost-effectiveness. [Expert Rev Pharmacoecon Outcomes Res.](https://www.ncbi.nlm.nih.gov/pubmed/23252357) 2012 Dec;12(6):745-64. doi: 10.1586/erp.12.67.
4. [Josephine K](https://www.ncbi.nlm.nih.gov/pubmed/?term=Josephine%20K%5BAuthor%5D&cauthor=true&cauthor_uid=28715726), [Josefine L](https://www.ncbi.nlm.nih.gov/pubmed/?term=Josefine%20L%5BAuthor%5D&cauthor=true&cauthor_uid=28715726), [Philipp D](https://www.ncbi.nlm.nih.gov/pubmed/?term=Philipp%20D%5BAuthor%5D&cauthor=true&cauthor_uid=28715726), [David E](https://www.ncbi.nlm.nih.gov/pubmed/?term=David%20E%5BAuthor%5D&cauthor=true&cauthor_uid=28715726), [Harald B](https://www.ncbi.nlm.nih.gov/pubmed/?term=Harald%20B%5BAuthor%5D&cauthor=true&cauthor_uid=28715726). Internet- and mobile-based depression interventions for people with diagnosed depression: a systematic review and meta-analysis. J Affect Disord 2017;223:28-40. doi: 10.1016/j.jad.2017.07.021.
5. Karyotaki E, Ebert DD, Donkin L, Riper H, et al. Do guided Internet-based interventions result in clinically relevant changes for patients with depression? An individual participant data meta-analysis Clin Psychol Rev 2018;63:80-92. doi: 10.1016/j.cpr.2018.06.007.
6. Karyotaki E, Riper H, Twisk J, et al. Efficacy of Self-guided Internet-Based Cognitive Behavioral Therapy in the Treatment of Depressive Symptoms: A Meta-analysis of Individual Participant Data. JAMA Psych. 2017;74(4):351-359. doi: 10.1001/jamapsychiatry.2017.0044
7. Richards D, Richardson T. Computer-based psychological treatments for depression: a systematic review and meta-analysis. Clin Psychol Rev 2012;32(4):329-342. doi: 10.1016/j.cpr.2012.02.004.
8. Twomey C, O'Reilly G, Meyer B. Effectiveness of an individually-tailored computerised CBT programme (Deprexis) for depression: A meta-analysis. Psychiatry Research 2017;256:371-377. doi: 10.1016/j.psychres.2017.06.081.
9. Wahle F, Bollhalder L, Kowatsch T, Fleisch E. Toward the Design of Evidence-Based Mental Health Information Systems for People With Depression: A Systematic Literature Review and Meta-Analysis. J Med Internet Res. 2017 May 31;19(5):e191. doi: 10.2196/jmir.7381.
10. Wright JH, Owen JJ, Richards D et al. Computer-assisted cognitive-behavior therapy for depression: A systematic review and meta-analysis, J Clin Psychiatry. 2019;80(2)18r12188, doi:10.3088/JXP.`8r12188

11. [Olthuis JV](https://www.ncbi.nlm.nih.gov/pubmed/?term=Olthuis%20JV%5BAuthor%5D&cauthor=true&cauthor_uid=26968204), [Watt MC](https://www.ncbi.nlm.nih.gov/pubmed/?term=Watt%20MC%5BAuthor%5D&cauthor=true&cauthor_uid=26968204), [Bailey K](https://www.ncbi.nlm.nih.gov/pubmed/?term=Bailey%20K%5BAuthor%5D&cauthor=true&cauthor_uid=26968204), [Hayden JA](https://www.ncbi.nlm.nih.gov/pubmed/?term=Hayden%20JA%5BAuthor%5D&cauthor=true&cauthor_uid=26968204), [Stewart SH](https://www.ncbi.nlm.nih.gov/pubmed/?term=Stewart%20SH%5BAuthor%5D&cauthor=true&cauthor_uid=26968204). Therapist-supported Internet cognitive behavioural therapy for anxiety disorders in adults. Cochrane Database Syst Rev 2016;3:CD011565. doi: 10.1002/14651858.

12. [Kampmann IL](https://www.ncbi.nlm.nih.gov/pubmed/?term=Kampmann%20IL%5BAuthor%5D&cauthor=true&cauthor_uid=27376634), [Emmelkamp PM](https://www.ncbi.nlm.nih.gov/pubmed/?term=Emmelkamp%20PM%5BAuthor%5D&cauthor=true&cauthor_uid=27376634), [Morina N](https://www.ncbi.nlm.nih.gov/pubmed/?term=Morina%20N%5BAuthor%5D&cauthor=true&cauthor_uid=27376634). Meta-analysis of technology-assisted interventions for social anxiety disorder. J Anxiety Disord 2016;42:71-84. doi: 10.1016/j.janxdis.2016.06.007.

13. Sijbrandij M, Kunovski I, Cuijpers P. Effectiveness of Internet-delivered cognitive behavioral therapy for posttraumatic stress disorder: a systematic review and meta-analysis. Depress Anxiety 2016;33(9):783–791. doi: 10.1002/da.22533.

14. Black N, Mullan B, Sharpe L. Computer-delivered interventions for reducing alcohol consumption: Meta-analysis and meta-regression using behaviour change techniques and theory. Health Psychol Rev 2016;7199(April):1–33. doi: 10.1080/17437199.2016.1168268.

15. [Boumparis N](https://www.ncbi.nlm.nih.gov/pubmed/?term=Boumparis%20N%5BAuthor%5D&cauthor=true&cauthor_uid=28295758), [Karyotaki E](https://www.ncbi.nlm.nih.gov/pubmed/?term=Karyotaki%20E%5BAuthor%5D&cauthor=true&cauthor_uid=28295758), [Schaub MP](https://www.ncbi.nlm.nih.gov/pubmed/?term=Schaub%20MP%5BAuthor%5D&cauthor=true&cauthor_uid=28295758), [Cuijpers P](https://www.ncbi.nlm.nih.gov/pubmed/?term=Cuijpers%20P%5BAuthor%5D&cauthor=true&cauthor_uid=28295758), [Riper H](https://www.ncbi.nlm.nih.gov/pubmed/?term=Riper%20H%5BAuthor%5D&cauthor=true&cauthor_uid=28295758).Internet interventions for adult illicit substance users: a meta-analysis. Addiction 2017;112(9):1521-1532. doi: 10.1111/add.13819.

16. Kaner EFS, Beyer FR, Garnett C, et al. Personalised digital interventions for reducing hazardous and harmful alcohol consumption in community-dwelling populations. Cochrane Database of Systematic Reviews 2017; 9:CD011479. doi: 10.1002/14651858.CD011479.pub2.

17. [Prosser T](https://www.ncbi.nlm.nih.gov/pubmed/?term=Prosser%20T%5BAuthor%5D&cauthor=true&cauthor_uid=29452058), [Gee KA](https://www.ncbi.nlm.nih.gov/pubmed/?term=Gee%20KA%5BAuthor%5D&cauthor=true&cauthor_uid=29452058), [Jones F](https://www.ncbi.nlm.nih.gov/pubmed/?term=Jones%20F%5BAuthor%5D&cauthor=true&cauthor_uid=29452058). A meta-analysis of effectiveness of E-interventions to reduce alcohol consumption in college and university students. [J Am Coll Health.](https://www.ncbi.nlm.nih.gov/pubmed/?term=Prosser+T%2C+Gee+KA) 2018;66(4):292-301. doi: 10.1080/07448481.2018.1440579.

18. Riper H, Blankers M, Hadiwijaya H, et al. Effectiveness of guided and ungided low-intensity Internet interventions for adult alcohol misuse: PLoS ONE 2014;9(6):e99912. doi: 10.1371/journal.pone.0099912.

19. Tait RJ, Spijkerman R, Riper H. Internet and computer based interventions for cannabis use: A meta-analysis. Drug and Alcohol Dependence 2013;133(2):295-304. doi: 10.1016/j.drugalcdep.2013.05.012.

20. Aardoom JJ, Dingemans AE, Spinhoven P, Van Furth EF. Treating eating disorders over the Internet: a systematic review and future research directions. Int J Eat Disord 2013;46(6):539-552. doi:10.1002/eat.22135.

21. Anastasiadou D, Folkvord F, Lupianez-Villanueva F. A systematic review of mHealth interventions for the support of eating disorders. Eur Eat Disord Rev. 2018;26(5):394-416. doi: 10.1002/erv.2609.

22. Fairburn CG, Murphy R. Treating eating disorders using the Internet. Curr Opin Psychiatry. 2015;28(6):461-7. doi: 10.1097/YCO.0000000000000195.

23. Loucas CE, Fairburn CG, Whittington C, Pennant ME, Stockton S, Kendall T. E-therapy in the treatment and prevention of eating disorders: a systematic review and meta-analysis. Behav Res Ther 2014;63:122-131. doi:10.1016/j.brat.2014.09.011.

24. Melioli T, Bauer S, Franko DL, et al. Reducing eating disorder symptoms and risk factors using the Internet: a meta-analytic review. Int J Eat Disord 2016;49(1):19-31. doi:10.1002/eat.22477.

25. Pittock A, Hodges L, Lawrie SM. The effectiveness of Internet-delivered cognitive behavioural therapy for those with bulimic symptoms: a systematic review: A review of iCBT treatment for bulimic symptoms. BMC Res Notes 2018;11(1):748. doi: 10.1186/s13104-018-3843-2.

26. Schlegl S, Burger C, Schmidt L, Herbst N, Voderholzer U. The potential of technology-based psychological interventions for anorexia and bulimia nervosa: a systematic review and recommendations for future research. J Med Internet Res 2015;17(3):e85. doi:10.2196/jmir.3554.
